# Psychological and social factors influencing quality of life during treatment in active breast cancer patients and psychological interventions: a systematic review

**DOI:** 10.3389/fpsyg.2026.1843805

**Published:** 2026-07-27

**Authors:** Noa Gay Rúa, María Rueda-Extremera, María Cantero-García

**Affiliations:** Facultad de Psicología y Ciencias de la Salud, Universidad a Distancia de Madrid, Madrid, Spain

**Keywords:** breast cancer, oncological treatment, psychological factors, psychological intervention, quality of life

## Abstract

Breast cancer remains one of the most prevalent oncological diseases among women, with its treatment exerting a substantial impact not only on physical health but also on psychological and social well-being. The present systematic review aims to identify the psychological and social factors that influence the quality of life (QoL) of women undergoing active breast cancer treatment. Additionally, it evaluates a range of psychological interventions designed to improve emotional well-being and compares their effectiveness in addressing specific psychosocial needs. This systematic review was conducted in accordance with the PRISMA 2020 guidelines. A comprehensive literature search was carried out across multiple databases—including Web of Science, Scopus, Dialnet, and ProQuest—between March 1 and March 27, 2025. The final sample comprised 15 studies published between 2014 and 2025, encompassing a total of 1,375 participants. The findings indicate that psychological interventions such as cognitive-behavioural therapy (CBT), mindfulness-based interventions, acceptance and commitment therapy (ACT), and behavioural activation contribute to significant improvements in emotional well-being and, in many cases, overall quality of life. However, several methodological limitations were identified across the reviewed studies, most notably the absence of long-term follow-up assessments and substantial heterogeneity in the measurement instruments employed. These limitations restrict the comparability of findings and underscore the need for more rigorous and standardized research designs. In conclusion, psychological interventions constitute a key component in the comprehensive management of breast cancer. Their systematic integration into healthcare protocols has the potential to markedly enhance patients’ quality of life, supporting a more holistic and person-centered approach to oncological care.

## Introduction

Breast cancer remains the most frequently diagnosed cancer among women worldwide and continues to represent a major public health challenge. Beyond its physical consequences, breast cancer and its treatment can substantially affect patients’ psychological and social well-being. Women undergoing active treatment often experience fatigue, pain, treatment-related side effects, uncertainty regarding treatment response, concerns about disease progression, changes in body image, and disruptions to family, social, and occupational functioning [The National Cancer Institute (NCI), 2025].

These challenges may have a profound impact on quality of life (QoL), particularly during active treatment, when patients must simultaneously cope with the physical demands of treatment and the emotional burden associated with cancer. QoL is generally understood as a multidimensional construct encompassing physical, psychological, and social well-being (Bautista-Rodriguez, 2017). Given the focus of the present review, particular attention is paid to its psychological and social dimensions, including emotional distress, coping processes, interpersonal relationships, social support, and adaptation to illness.

Among the psychological factors influencing QoL, emotional distress is particularly relevant during active treatment. Anxiety, depressive symptoms, fear of recurrence, uncertainty, and difficulties adjusting to treatment demands are common among women with breast cancer. Reported prevalence rates vary substantially depending on the population studied and the assessment methods employed, but emotional difficulties consistently represent one of the strongest predictors of reduced quality of life (Fereidouni et al., 2024). Early identification of psychological symptoms and timely psychosocial support are therefore considered essential components of comprehensive cancer care.

Coping strategies also play a central role in psychological adjustment to breast cancer. Adaptive strategies such as acceptance, emotional expression, information seeking, and reliance on social support have been associated with better psychological outcomes, whereas avoidant coping patterns are generally linked to greater distress (Cerquera et al., 2017). In addition, symptom burden—including fatigue, pain, sleep disturbances, and concerns regarding the future—can further compromise quality of life during treatment (Font et al., 2004; Singh et al., 2024; Rey, 2017).

Psychological care constitutes a critical component of comprehensive cancer management. Among the psychosocial interventions most frequently applied in oncology are cognitive-behavioural therapy (CBT), mindfulness-based interventions, Acceptance and Commitment Therapy (ACT), behavioural activation, positive psychology approaches, and other supportive interventions aimed at enhancing coping and emotional adjustment. These approaches seek to reduce emotional distress, strengthen psychological resources, and improve patients’ adaptation to treatment-related challenges. Previous research has reported promising effects on anxiety, depression, coping skills, and emotional well-being, particularly among women undergoing active treatment (Zhang et al., 2022; Greeley et al., 2025; Restrepo and León, 2020; González de León et al., 2022).

Beyond structured psychotherapeutic approaches, integrative body–mind and stress-management interventions—such as yoga-based programs and guided self-administered techniques—have been incorporated increasingly into oncology settings. These interventions combine physiological regulation strategies (e.g., breathing, relaxation) with attentional and emotional modulation and are frequently framed within complementary or supportive care models (Takemura et al., 2025). Although yoga-based interventions and mindfulness-based approaches share certain components, such as attentional focus, body awareness, and breathing regulation, they differ in their primary therapeutic emphasis. Mindfulness-based interventions are generally designed to cultivate non-judgmental awareness of present-moment experiences through formal meditation practices, whereas yoga-based interventions integrate physical postures, movement, and physiological regulation as core therapeutic components. For this reason, yoga-based programs were considered a separate intervention category in the present review, while acknowledging potential conceptual overlap between both approaches.

Distinguishing conceptually among these families of interventions is essential for identifying potential differences in their mechanisms of action and clinical outcomes, particularly during the active phase of treatment.

Social support constitutes a fundamental protective factor against the adverse psychological and social consequences of cancer. Robust social networks have been shown to buffer illness-related stress and promote adaptive coping responses (Revenson et al., 1983). Within these networks, family support—particularly from spouses or partners—emerges as the most accessible, consistent, and valued source of assistance for patients navigating the disease trajectory (Chilon-Huaman et al., 2023).

Despite the established relevance of social support, Álvarez and Pérez (2024) emphasizes that quality of life (QoL) remains a complex and inherently subjective construct for which no single, universally accepted definition exists. Patients’ experiences vary widely, and comprehensive cancer care must consider the interplay of physical, emotional, and social dimensions. To evaluate QoL reliably, validated assessment tools are therefore indispensable. Instruments such as the EORTC QLQ-C30 have been widely employed to measure multiple domains of health-related QoL (Álvarez and Pérez, 2024; Sanz, 2016; Rey, 2017). Higher levels of physical functioning, appetite, and overall QoL have been associated with longer survival, whereas elevated pain levels have consistently predicted poorer clinical outcomes.

An important consideration is that the active treatment phase represents a unique clinical context that differs substantially from cancer survivorship. During chemotherapy, radiotherapy, surgery, or hormone therapy, patients frequently experience fatigue, pain, treatment-related side effects, cognitive difficulties, uncertainty regarding treatment response, and concerns about disease progression. At the same time, many women face disruptions in family, social, and occupational functioning, which may further compromise psychological well-being and quality of life. These circumstances can influence not only the intensity of psychosocial needs but also the feasibility and effectiveness of psychological interventions. Ongoing treatment schedules, physical symptom burden, and reduced energy levels may limit participation in psychosocial programs, while simultaneously increasing the need for emotional support and coping resources. Consequently, findings derived from survivorship populations cannot be assumed to generalize directly to women undergoing active treatment, highlighting the importance of examining this population separately.

Although an increasing number of systematic reviews have investigated psychosocial interventions in breast cancer populations, several critical gaps remain unaddressed (King et al., 2024; Kemp et al., 2022; Guarino et al., 2020; Azizi et al., 2024). First, many reviews have centred primarily on survivorship or post-treatment adjustment, rather than focusing exclusively on women undergoing active oncological treatment—a period marked by acute symptom burden, heightened emotional vulnerability, and pronounced treatment-related uncertainty. Second, prior reviews frequently merge heterogeneous treatment phases within a single analytical framework, thereby limiting the ability to derive clinically meaningful, phase-specific conclusions. Third, only a limited number of studies have conducted comparative analyses of psychological interventions including cognitive-behavioural therapy (CBT), mindfulness-based interventions, Acceptance and Commitment Therapy (ACT), behavioural activation, positive psychology interventions, yoga-based interventions, and self-administered stress-management. Finally, emotional outcomes such as anxiety and depression are often examined without sufficient differentiation from broader, multidimensional QoL constructs, hindering conceptual clarity. Consequently, there remains a need to synthesize the available evidence specifically in women undergoing active breast cancer treatment in order to determine which psychosocial interventions are most effective, for whom they are most beneficial, and under what treatment conditions they can be successfully implemented.

The present review addresses these gaps by concentrating exclusively on the active treatment phase and by systematically comparing the effects of different psychological interventions across emotional outcomes and wider QoL domains. In light of the available evidence, psychological and social factors appear to play a central role not only in shaping how women cope with the diagnosis of breast cancer but also in influencing their adaptation throughout the treatment process.

Accordingly, the objectives of this systematic review are threefold: (1) to identify the primary psychological and social factors that influence the QoL of women undergoing active breast cancer treatment; (2) to comparatively evaluate the effectiveness of different psychological interventions—including CBT, mindfulness-based interventions, ACT, behavioural activation, positive psychology approaches, yoga-based programs, and other supportive interventions—on emotional well-being and QoL outcomes; and (3) to inform clinical practice by clarifying which psychosocial domains respond most robustly to specific therapeutic approaches during the active treatment period.

## Method

This systematic review was conducted in accordance with the Preferred Reporting Items for Systematic Reviews and Meta-Analyses (PRISMA 2020) guidelines, which provide a standardized framework to ensure methodological rigor, transparency, and reproducibility in evidence synthesis (Urrútia and Bonfill, 2010).

The review included empirical studies published between January 2014 and March 2025 in English, Spanish, or Catalan that examined psychological and social factors influencing the quality of life of women undergoing active treatment for breast cancer. The review protocol and methodological procedures were prospectively documented and made publicly accessible through the Open Science Framework (OSF), which enhances transparency and allows external verification of the review process.

Although the protocol was not registered in PROSPERO, all methodological decisions—including eligibility criteria, search strategy parameters, and analytic procedures—were predefined prior to data extraction in order to minimize potential bias and increase methodological transparency.

Because the present review is based exclusively on previously published studies and does not involve the collection or handling of identifiable human participant data, ethical approval was not required.

### Eligibility criteria

To clearly define the scope of the review, the PICO(S) framework was applied (see Table 1). The inclusion criteria were established as follows: empirical studies employing randomized controlled trials, correlational designs, quasi-experimental designs, or experimental methodologies;studies involving women aged 18 years or older;research focused on participants undergoing active oncological treatment for breast cancer (e.g., chemotherapy, radiotherapy, hormone therapy, or surgical treatment);publications written in English, Spanish, or Catalan.

**Table 1:** Description of the PICO process.

PICO Component	Eligibility Criteria
P	Adult female patients with breast cancer undergoing active treatment (chemotherapy, radiotherapy, immunotherapy, surgery, or other oncological therapies).
I	Psychological and social factors associated with quality of life (QoL).
C	Different coping strategies or therapies.
O	Impact on quality of life.
(s)	Controlled clinical trials, quasi-experimental studies, and cohort studies.

Exclusion criteria were defined to ensure methodological consistency and thematic relevance and included the following: theses, books, and monographs; articles published prior to 2014; studies conducted outside the European Union or the Americas; research specifically addressing the impact of COVID-19; studies involving samples receiving palliative care (see Table 2).

**Table 2:** Selection criteria.

Inclusion criteria	Exclusion criteria
ECAS, correlational or experimental studies	Articles and studies conducted outside the Europe Union or the Americas
Written in English, Spanish, or Catalan	Theses, books, monographs
Women over 18 years old	Articles published before 2014
Women undergoing oncological treatment	Articles discussing COVID-19
Women with breast cancer	Populations receiving palliative care

These criteria ensured the selection of methodologically robust and conceptually aligned studies addressing psychological and social factors influencing the quality of life of women undergoing active treatment for breast cancer. The review focused exclusively on women undergoing active breast cancer treatment because this phase is characterised by unique physical, emotional, and practical challenges that may influence both psychosocial needs and intervention effectiveness.

The review focused on studies conducted in the European Union and the Americas in order to reduce contextual heterogeneity related to healthcare systems, psychosocial oncology services, and cultural differences in psychological intervention delivery. Limiting the geographical scope allowed greater comparability across healthcare settings and intervention frameworks.

### Information sources and search strategy

The literature search was conducted across four electronic databases—Web of Science, Scopus, Dialnet, and ProQuest—between March 1 and March 27, 2025, and studies published after this period were not considered. A structured search strategy was developed for each database using combinations of controlled vocabulary and free-text terms related to breast cancer, psychological variables, and quality of life.

Search terms were applied in both Spanish and English and included combinations such as:

*Spanish terms:* psicooncología, factores psicológicos, calidad de vida, ansiedad, depresión, tratamiento activo, quimioterapia, en tratamiento, cáncer de mama, supervivientes de cáncer, postratamiento, remisión.

*English terms:* cancer patients, oncology patients, neoplasm, psychological factors, anxiety, depression, quality of life, active treatment, undergoing treatment, receiving chemotherapy, cancer survivors, post-treatment, remission.

Boolean operators (“AND,” “OR,” and “NOT”) were used to combine search terms and refine results within each database. Truncation and database-specific filters were applied when available in order to optimize search sensitivity and precision. The complete search strategy used for each database is provided in Table 3.

**Table 3:** Sources and information search strategies.

Database	Search equation	Number of references found
Dialnet	psicooncología AND factores psicológicos *Filters*: 2014-present	49
	
Scopus	“cancer patients” OR “oncology patients” OR “neoplasms” AND “psychological factors” OR “anxiety” OR “depression” AND “quality of life” AND (“active treatment” OR “undergoing treatment” OR “receiving chemotherapy”) AND NOT (“cancer survivors” OR “post-treatment” OR “remission”) *Filters*: 2014-present	61
	
ProQuest	(“cancer patients” OR “oncology patients” OR “neoplasms”) AND (“psychological factors” OR “anxiety” OR “depression”) AND “quality of life” AND (“active treatment” OR “undergoing treatment” OR “receiving chemotherapy”) AND “breast cancer” NOT (“cancer survivors” OR “post-treatment” OR “remission”) *Filters*: 2014-present, English or Spanish, Scientific Journals	209
	
ProQuest	(“pacientes con cáncer” OR “pacientes oncológicas” OR “neoplasias”) AND (“factores psicológicos” OR “ansiedad” OR “depresión”) AND “calidad de vida” AND (“tratamiento activo” OR “en tratamiento” OR “quimioterapia”) AND “cáncer de mama” NOT (“supervivientes de cáncer” OR “postratamiento” OR “remisión”) *Filters*: 2014-present, English or Spanish, Scientific Journals	91
	
Web of Science	(“breast cancer” OR “mammary carcinoma”) AND (“psychosocial factors” OR “social support” OR “coping” OR “psychological distress” OR “mental health” OR “emotional well-being”) AND (“quality of life”) AND (“treatment” OR “chemotherapy” OR “radiotherapy” OR “oncological treatment”)) *Filters*: 2014-present, English or Spanish, Scientific Journals Open Access Countries in Europe and America	502

### Study selection

**Figure 1:**
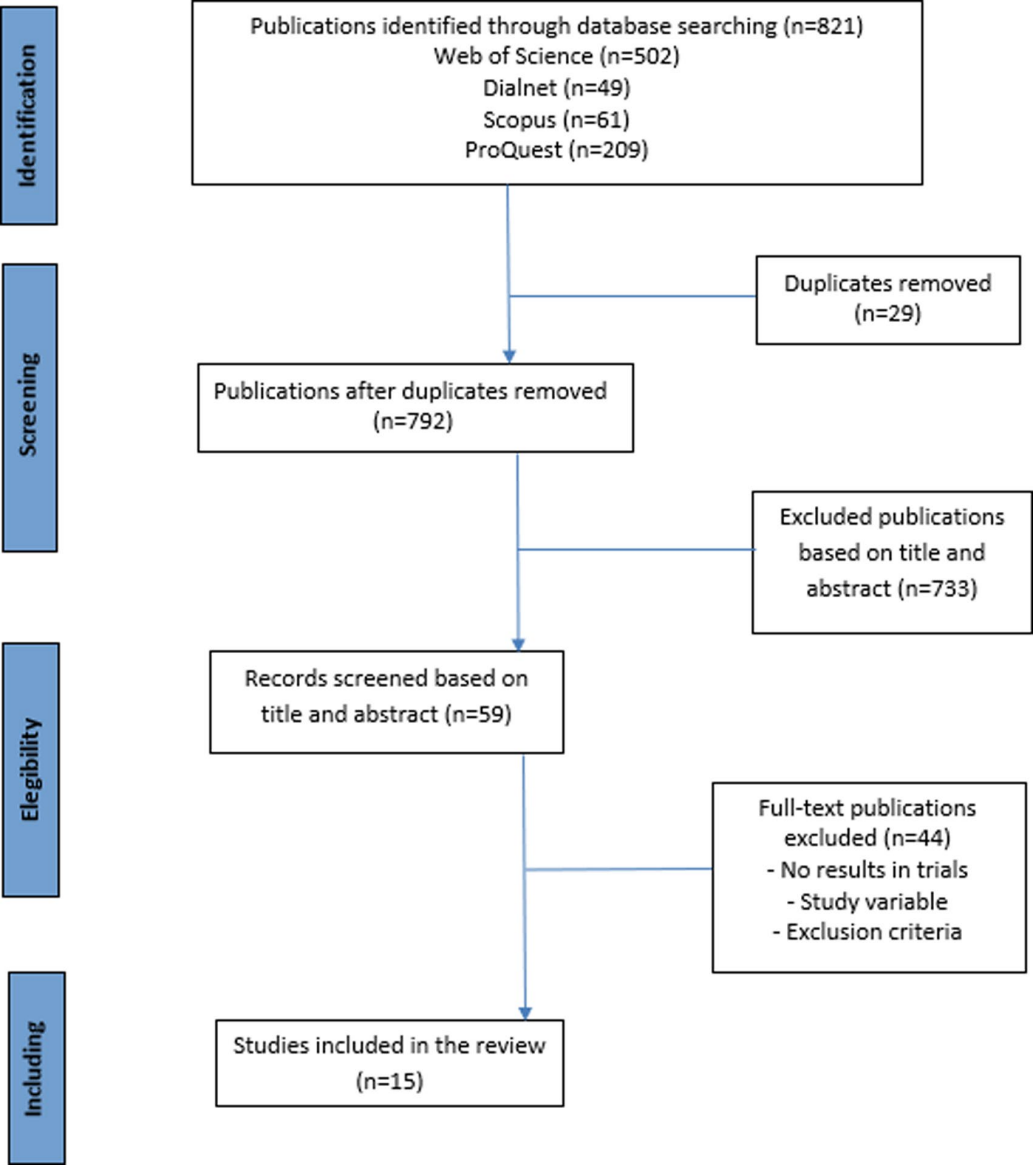
PRISMA-based systematic review process flowchart.

The initial search yielded 821 records (*N* = 821). After removing duplicates (*N* = 29) and applying the automated filters available in the databases, titles and abstracts of 792 publications (*N* = 792) were screened according to the inclusion criteria, resulting in 59 articles (*N* = 59) selected for full-text review. Following a comprehensive assessment of methodological and thematic eligibility, 44 studies were excluded for not meeting the predefined criteria, leaving a final sample of 15 studies (*N* = 15) for inclusion in the review (see PRISMA flowchart in Figure 1). Study selection was conducted in two stages following PRISMA recommendations. The screening process was performed independently by two reviewers. Any disagreements regarding study inclusion were resolved through discussion and consensus. When consensus could not be reached, a third reviewer was consulted to adjudicate the final decision.

### Data extraction and analysis

Data extraction was conducted using a standardized data extraction specifically designed for this review.

The following information was collected from each study: author and year of publication, country, study design, sample characteristics, type of psychological intervention, outcome measures, and main results related to quality of life and psychosocial variables. Data extraction was performed by one reviewer and subsequently verified by a second reviewer to ensure accuracy and completeness.

Study selection and data extraction were carried out independently by two reviewers using a predefined extraction template, and any discrepancies were resolved through discussion and consensus; when necessary, a third reviewer adjudicated disagreements.

Extracted information included study design, sample characteristics, intervention type, outcome measures, and principal findings. Specifically, data were collected on participant characteristics (particularly age), psychological and social variables associated with quality of life (including anxiety, depression, and stress), and intervention characteristics. For synthesis purposes, interventions were classified according to their primary therapeutic orientation: cognitive-behavioural therapy (CBT), mindfulness-based interventions, contextual cognitive-behavioural approaches (including Acceptance and Commitment Therapy and Behavioural Activation), positive psychology interventions, yoga-based supportive interventions, and self-administered stress-management programs.

The methodological quality of randomized controlled trials was assessed using the Physiotherapy Evidence Database (PEDro) scale, which evaluates both internal and external validity, and the Cochrane Risk of Bias Tool (Higgins and Green, 2011) was used to estimate potential sources of bias and assess the reliability of the evidence generated by each study.

Given the marked heterogeneity across studies in terms of intervention type, duration, delivery format (individual, group, or self-administered), outcome measures, and research designs, it was determined *a priori* that a quantitative meta-analysis would only be conducted if sufficient methodological homogeneity was observed. Following study selection, the degree of clinical and methodological heterogeneity was considered too substantial to support meaningful effect-size aggregation. Consequently, a structured narrative synthesis was undertaken to identify patterns of effectiveness, assess consistency across studies, and examine differences in outcomes across emotional and broader quality-of-life domains.

## Results

### Study characteristics

This review comprises a total of 15 studies published between 2014 and 2025, including randomized controlled trials (*n* = 6), quasi-experimental studies (*n* = 3), pre-experimental studies (*n* = 2), descriptive studies (*n* = 3), and one mixed-methods study (*n* = 1). Across these investigations, the most frequently assessed variables were depression (*n* = 12), anxiety (*n* = 11), psychological well-being (*n* = 13), and quality of life (QoL) (*n* = 7), reflecting a strong emphasis on emotional and psychosocial outcomes during active breast cancer treatment. To improve comparability across studies, intervention outcomes are synthesized according to outcome domain, with intervention types discussed within each domain.

### Sample

The total sample comprised 1,375 women diagnosed with breast cancer who were undergoing various stages of active treatment, including chemotherapy, radiotherapy, hormone therapy, or surgery. Across all fifteen studies, participants were recruited through non-probability sampling methods, predominantly convenience sampling. The typical age range of the samples fell between 30 and 70 years. Sample sizes varied substantially, from as few as 6 participants in Belber-Gómez et al. (2018) to 240 participants in Hoogland et al. (2018), reflecting notable heterogeneity in study scope and statistical power.

### Interventions

All included studies aimed to evaluate quality of life directly or to assess psychosocial factors influencing quality of life during active breast cancer treatment. Interventions encompassed a range of psychological and psychosocial approaches, including Cognitive-Behavioural Therapy (CBT), mindfulness-based interventions, Acceptance and Commitment Therapy (ACT), Behavioural Activation, positive psychology programmes, yoga-based interventions, and self-administered stress-management programmes.

The included studies assessed a wide range of psychological and quality-of-life outcomes. However, primary and secondary outcomes were not consistently predefined or reported across studies, limiting direct comparisons and increasing methodological heterogeneity.

To facilitate interpretation and comparison, interventions were grouped according to their primary therapeutic orientation: Cognitive-Behavioural Therapy (CBT), Mindfulness-Based Interventions, Contextual Cognitive-Behavioural and Positive Psychology Interventions (including ACT and Behavioural Activation), Yoga-Based Interventions, and Self-Administered Stress-Management Programmes. Details of the interventions and outcome measures are presented in Table 4.

**Table 4:** Classification of interventions and study variables.

Publications	Variables			
	ANS	DEP	QoL	Well-being
Ardizzone et al. (2022)				
Belber-Gómez et al. (2018)				
Bisseling et al. (2017)				
Cerezo et al. (2014)				
Chandwani et al. (2014)				
Franco et al. (2020)				
González et al. (2015)				
Hoogland et al. (2018)				
Kenne et al. (2017)				
Martínez-Cuervo et al. (2020)				
Micheletti et al. (2022)				
Mondo et al. (2015)				
Moraga et al. (2020)				
Vila et al. (2016)				
Villoria et al. (2020)				

ANS = Anxiety; DEP = Depression; QoL = Quality of Life and Well-being (emotional, physical, and social).

Green shading indicates that the study assessed the corresponding outcome. White cells indicate that the outcome was not evaluated or not reported.

### Cognitive-behavioural therapy (CBT)

Four studies examined the effectiveness of individual or group Cognitive-Behavioural Therapy (CBT), consistently evaluating anxiety and depression alongside additional psychological and functional variables.

Across the reviewed studies, CBT interventions showed the most consistent reductions in anxiety and depressive symptoms among women undergoing active breast cancer treatment.

Martínez-Cuervo et al. (2020), the only study employing group-based CBT, evaluated its impact on depression, anxiety, and affective-autonomous functioning through a program incorporating psychoeducation, relaxation techniques, and cognitive restructuring. This study reported large effect sizes, with significant decreases in anxiety (*F* = 49.66; *p* < 0.001; partial η^2^ = 0.254) and depression (*F* = 55.24; *p* < 0.001; partial η^2^ = 0.274).

Moraga et al. (2020) assessed the effectiveness of individual CBT on anxiety, depression, and coping strategies by comparing three groups: CBT with psychoeducation (G1), counseling with psychoeducation (G2), and a control group. Significant pre-post reductions were observed in anxiety (t = 4.61; *p =* 0.001) and depression (t = 2.99; *p =* 0.013).

Ardizzone et al. (2022) implemented three individual psychoeducational CBT sessions and compared outcomes with a control group receiving a self-guided informational booklet. The intervention group showed significant reductions in anxiety (t = 3.72; *p =* 0.000) and depression (t = 3.13; *p =* 0.003).

González et al. (2015) also reported a significant decrease in anxiety (t = −2.36; *p =* 0.027), although depression did not show statistically significant change (t = 1.84; *p =* 0.08); however, significant improvements appeared in the global HADS score (T = 2.81; *p =* 0.014).

### Contextual cognitive-behavioural approaches: ACT and behavioural activation

Positive psychology–based interventions were examined in two studies:

Across studies using positive psychology and ACT-based approaches, improvements were primarily observed in emotional regulation and behavioural functioning rather than in consistent reductions in anxiety or depression.

Vila et al. (2016) evaluated emotional states before and after a psychotherapy session and found significant reductions in sadness intensity (Z = −3.239; *p =* 0.001), nervousness (Z = −3.030; *p =* 0.002), fear (Z = −2.416; *p =* 0.016), and emotional disturbance (Z = −3.030; *p =* 0.002).

Cerezo et al. (2014) reported significant improvements in cognitive well-being, positive and negative affect, attention, and self-esteem (all *p* < 0.05) following a group intervention grounded in positive psychology.

Villoria et al. (2020), using behavioural activation, reported significant improvements in domestic activities (*p* = 0.005) and leisure participation (*p* = 0.05), although no improvements were observed in sexual activity or pleasurable activities.

Belber-Gómez et al. (2018), using ACT and mindfulness techniques in a qualitative framework, identified improvements in emotional validation, adaptive coping strategies, and perceived psychological support.

### Mindfulness-based therapies

Four studies investigated mindfulness-based interventions.

Overall, mindfulness-based interventions demonstrated moderately consistent improvements in emotional distress and psychological well-being, although results for anxiety and depression were heterogeneous.

Bisseling et al. (2017) examined mindfulness-based stress reduction (MBSR) and found no significant improvements in HADS scores (*p* = 0.512), nor in physical well-being (*p* = 0.31), emotional functioning (*p* = 0.14), or global QoL (*p* = 0.085).

In contrast, Kenne et al. (2017) reported significant reductions in global distress (*p* = 0.013) and psychological symptoms (*p* = 0.019), along with improved mental health (*p* = 0.001), coping strategies (*p* = 0.028), and mindfulness.

Mondo et al. (2015) documented significant reductions in depression (Z = −2.120; *p* = 0.034) and a marginal reduction in anxiety (Z = −1.807; *p* = 0.071), along with increases in mindfulness (*p* = 0.024).

Franco et al. (2020), using flow meditation, reported significant improvements in anxiety (Z = 2.68; *p* = 0.009), social avoidance (Z = 3.57; *p* = 0.001), depression (Z = 4.56; *p* < 0.001), resilience (Z = 2.97; *p* = 0.004), and self-esteem (Z = 5.06; *p* < 0.001).

### Yoga-based interventions

Two studies evaluated yoga-based interventions. Findings from yoga interventions were mixed and tended to show greater impact on physical outcomes than on psychological symptoms. Micheletti et al. (2022) observed short-term improvements in emotional functioning but no significant changes in global QoL or fatigue. Chandwani et al. (2014) reported improvements in physical QoL (*p* = 0.05), psychological distress (*p* = 0.04), and fatigue reduction (*p* = 0.02), although no significant improvements were found in mental health (*p* = 0.20) or sleep quality (*p* = 0.90).

### Self-administered interventions

Hoogland et al. (2018) evaluated the effectiveness of a self-administered stress-management program on anxiety, depression, stress, emotional well-being, and spiritual well-being. Participants in the intervention group received written materials describing deep-breathing exercises, progressive muscle relaxation accompanied by guided imagery, and positive affirmations, enabling them to practice these techniques independently. The control group, by contrast, received only an educational pamphlet. This design allowed the authors to assess the specific contribution of a self-guided psychological program to emotional and functional outcomes during active breast cancer treatment.

### Methodological quality results

Methodological quality was assessed using the PEDro scale (Table 5). None of the studies achieved the maximum possible score of 10. Twelve studies (Ardizzone et al., 2022; Martínez-Cuervo et al., 2020; Moraga et al., 2020; González et al., 2015; Bisseling et al., 2017; Franco et al., 2020; Kenne et al., 2017; Cerezo et al., 2014; Mondo et al., 2015; Micheletti et al., 2022; Chandwani et al., 2014; Hoogland et al., 2018) obtained a score of 6, indicating good methodological quality. The remaining three studies (Vila et al., 2016; Villoria et al., 2020; Belber-Gómez et al., 2018) scored 5, reflecting fair methodological quality.

**Table 5:** Methodological quality results.

PEDro scale												
Publications	1	2	3	4	5	6	7	8	9	10	11	Total score
Ardizzone et al. (2022)	Yes	Yes	No	Yes	No	No	No	Yes	Yes	Yes	Yes	6
Belber-Gómez et al. (2018)	Yes	No	No	Yes	No	No	No	Yes	Yes	Yes	Yes	5
Bisseling et al. (2017)	Yes	Yes	No	Yes	No	No	No	Yes	Yes	Yes	Yes	6
Cerezo et al. (2014)	Yes	Yes	No	Yes	No	No	No	Yes	Yes	Yes	Yes	6
Chandwani et al. (2014)	Yes	Yes	No	Yes	No	No	No	Yes	Yes	Yes	Yes	6
Franco et al. (2020)	Yes	Yes	No	Yes	No	No	No	Yes	Yes	Yes	Yes	6
González et al. (2015)	Yes	Yes	No	Yes	No	No	No	Yes	Yes	Yes	Yes	6
Hoogland et al. (2018)	Yes	Yes	No	Yes	No	No	No	Yes	Yes	Yes	Yes	6
Kenne et al. (2017)	Yes	Yes	No	Yes	No	No	No	Yes	Yes	Yes	Yes	6
Martínez-Cuervo et al. (2020)	Yes	Yes	No	Yes	No	No	No	Yes	Yes	Yes	Yes	6
Micheletti et al. (2022)	Yes	Yes	No	Yes	No	No	No	Yes	Yes	Yes	Yes	6
Moraga et al. (2020)	Yes	Yes	No	Yes	No	No	No	Yes	Yes	Yes	Yes	6
Mondo et al. (2015)	Yes	Yes	No	Yes	No	No	No	Yes	Yes	Yes	Yes	6
Vila et al. (2016)	Yes	No	No	Yes	No	No	No	Yes	Yes	Yes	Yes	5
Villoria et al. (2020)	Yes	No	No	Yes	No	No	No	Yes	Yes	Yes	Yes	5

1. The eligibility criteria were specified; 2. Subjects were randomly assigned to groups; 3. Allocation was concealed; 4. Groups were similar at baseline regarding the most important prognostic indicators; 5. All subjects were blinded; 6. All therapists administering the therapy were blinded; 7. All assessors who measured at least one key outcome were blinded; 8. Measurements for at least one key outcome were obtained from more than 85% of the subjects initially assigned to groups; 9. Results were reported for all subjects who received treatment or were assigned to the control group, or when this was not possible, data for at least one key outcome were analyzed using “intention-to-treat” analysis; 10. Statistical comparisons between groups were reported for at least one key outcome; 11. The study provides point measures and measures of variability for at least one key outcome.

### Risk of bias results

**Figure 2:**
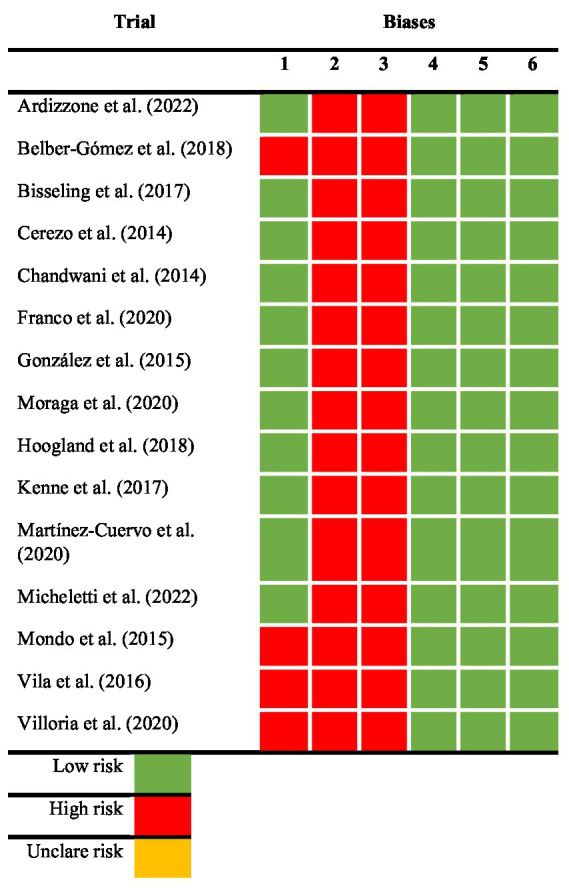
Results of risk of bias assessment. 1. Selection bias (random sequence generation); 2. Performance bias (blinding of participants and personnel); 3. Detection bias (blinding of outcome assessors); 4. Attrition bias; 5. Bias due to incomplete outcome data. 6. Reporting bias.

Risk of bias was assessed using the Cochrane risk of bias framework Cochrane risk of bias was assessed for all studies (Figure 2). Regarding selection bias, most studies presented a low risk due to the use of random allocation procedures and comparable baseline groups; however, Villoria et al. (2020), Belber-Gómez et al. (2018), Vila et al. (2016), and Mondo et al. (2015) did not implement adequate randomization methods, resulting in a high risk of bias in this domain. Performance bias was high across all studies, as blinding of participants and personnel was not feasible given the nature of psychological interventions. Detection bias was also high in all studies due to the absence of blinded outcome assessors. In contrast, attrition bias was low across the full set of studies, as attrition rates were clearly reported and analyses were conducted with complete cases. Incomplete outcome data bias was similarly low, with all studies providing sufficient justification for the outcomes presented. Reporting bias was assessed as low across all studies, indicating no evidence of selective reporting.

Overall, the global risk of bias was considered high, largely due to the inherent challenges of blinding in psychosocial intervention research. Furthermore, small sample sizes and widespread reliance on non-probability sampling procedures limit the generalizability of the findings and should be considered when interpreting intervention effectiveness. High, primarily due to the inherent impossibility of blinding participants and evaluators in psychological intervention research. This structural limitation may contribute to an overestimation of treatment effects. Furthermore, small sample sizes and the widespread use of non-probabilistic recruitment methods reduce the robustness and generalizability of the findings. These methodological constraints must therefore be taken into account when interpreting the effectiveness of the interventions included in this review.

Only four of the fifteen included studies explicitly prespecified a primary outcome [two studies (Chandwani et al., 2014; Hoogland et al., 2018) identified the Physical Component Summary (PCS) of the SF-36 as the primary endpoint, one study (Kenne et al., 2017) identified anxiety and depression assessed with the Hospital Anxiety and Depression Scale (HADS), and one (Martínez-Cuervo et al., 2020) identified depression and anxiety assessed with the Beck Depression Inventory (BDI) and Hamilton Anxiety Rating Scale (HAM-A)]. In two studies (Bisseling et al., 2017; Mondo et al., 2015), the presence of a predefined primary outcome was unclear, whereas the remaining nine studies evaluated multiple psychological outcomes without specifying a primary endpoint or distinguishing between primary and secondary outcomes. This lack of prespecification increases the potential for selective outcome reporting and complicates the interpretation of statistically significant findings.

Similarly, adjustment for multiple comparisons was reported in only one study (Martínez-Cuervo et al., 2020); the remaining studies performed multiple statistical tests across several outcomes and/or time points without reporting any strategy to control the family-wise type I error rate. Consequently, statistically significant findings should be interpreted with caution.

### Intervention effects

To facilitate comparison across studies, intervention effects were synthesized according to outcome domain and intervention type. Overall, improvements were more consistently observed in emotional outcomes (anxiety, depression, and psychological distress) than in multidimensional quality-of-life measures. Cognitive-behavioural interventions generally produced the most consistent reductions in emotional symptoms, whereas mindfulness-based, contextual, and positive psychology interventions showed more variable effects. Yoga-based and self-administered interventions demonstrated more heterogeneous outcomes.

### Anxiety and depression

Anxiety and depression were among the most frequently evaluated outcomes, appearing in 11 and 12 studies, respectively. Across intervention categories, reductions in emotional distress were generally more consistent than improvements in broader quality-of-life indicators.

Cognitive-behavioural therapy (CBT).
Four studies evaluated CBT interventions during active breast cancer treatment.

Martínez-Cuervo et al. (2020) reported large effect sizes following a group-based CBT programme, with significant reductions in anxiety (*F* = 49.66; *p* < 0.001; partial η^2^ = 0.254) and depression (*F* = 55.24; *p* < 0.001; partial η^2^ = 0.274).

Moraga et al. (2020) found significant pre-post reductions in anxiety (t = 4.61; *p* = 0.001) and depression (t = 2.99; *p* = 0.013) following individual CBT combined with psychoeducation.

Ardizzone et al. (2022) observed significant decreases in anxiety (t = 3.72; *p* = 0.000) and depression (t = 3.13; *p* = 0.003) after three psychoeducational CBT sessions.

González et al. (2015) reported significant improvements in anxiety (t = −2.36; *p* = 0.027), although changes in depression did not reach statistical significance (t = 1.84; *p* = 0.08); however, significant improvements were observed in overall HADS scores (T = 2.81; *p* = 0.014).

Among the interventions reviewed, CBT appeared to demonstrate the most consistent reductions in anxiety and depressive symptoms. However, this conclusion should be interpreted cautiously given the limited number of studies, methodological heterogeneity, relatively small samples, inconsistent reporting of effect sizes, and overall risk of bias.

### Mindfulness-based interventions

Although mindfulness-based approaches represent one of the most extensively studied intervention modalities in psycho-oncology, only four studies met the eligibility criteria for inclusion in this review. This finding appears to reflect the restriction to women undergoing active treatment, as many mindfulness studies have been conducted during survivorship or after treatment completion.

Bisseling et al. (2017) found no significant improvements in HADS scores (*p* = 0.512). In contrast, Kenne et al. (2017) reported significant reductions in global distress (*p* = 0.013), psychological symptoms (*p* = 0.019), and improvements in mental health (*p* = 0.001).

Mondo et al. (2015) observed significant reductions in depression (Z = −2.120; *p* = 0.034) and a marginal reduction in anxiety (Z = −1.807; *p* = 0.071).

Franco et al. (2020) reported significant improvements in anxiety (Z = 2.68; *p* = 0.009), social avoidance (Z = 3.57; *p* = 0.001), and depression (Z = 4.56; *p* < 0.001).

Overall, mindfulness-based interventions demonstrated moderate benefits for emotional distress and psychological adjustment, although findings were heterogeneous across studies.

### Contextual cognitive-behavioural and positive psychology interventions

Vila et al. (2016) reported significant reductions in sadness intensity (Z = −3.239; *p* = 0.001), nervousness (Z = −3.030; *p* = 0.002), fear (Z = −2.416; *p* = 0.016), and emotional disturbance (Z = −3.030; *p* = 0.002).

Cerezo et al. (2014) observed significant improvements in cognitive well-being, positive affect, negative affect, attention, and self-esteem (all *p* < 0.05).

Villoria et al. (2020) did not report significant reductions in anxiety or depression.

Belber-Gómez et al. (2018) identified qualitative improvements in emotional validation, adaptive coping, and perceived psychological support.

Collectively, these interventions appeared to exert their strongest effects on emotional adaptation, coping, and behavioural functioning rather than on clinically significant reductions in anxiety and depression.

### Yoga-based and self-administered interventions

Hoogland et al. (2018) reported improvements in stress management (*p* < 0.05), but found no significant differences in anxiety, depression, or cancer-related distress.

Chandwani et al. (2014) observed improvements in psychological distress (*p* = 0.04), whereas Micheletti et al. (2022) reported only short-term emotional benefits.

Overall, yoga-based and self-administered interventions appeared to provide selective benefits but demonstrated less consistent effects on anxiety and depression than CBT or mindfulness-based approaches.

### Quality of life and well-being

An important finding across studies was that improvements in emotional symptoms were generally more consistent than improvements in multidimensional quality-of-life outcomes. Positive effects on QoL often occurred in specific domains rather than in global QoL scores.

### Cognitive-behavioural therapy (CBT)

González et al. (2015) reported significant improvements in physical health (*p* = 0.016) and interpersonal relationships (*p* = 0.048), although no significant changes were observed in psychological health (*p* = 0.099) or environmental well-being (*p* = 0.567).

Ardizzone et al. (2022) found significant pre-post improvements in global QoL (*p* < 0.001) and in physical, emotional, and social functioning (all *p* < 0.001). Significant reductions were also observed in fatigue, nausea, pain, dyspnoea, and insomnia (all *p* < 0.001), although no significant between-group differences were reported.

Taken together, CBT interventions appeared capable of improving selected domains of quality of life, although evidence for consistent improvements in global QoL remains limited.

### Mindfulness-based interventions

Bisseling et al. (2017) did not observe significant improvements in physical well-being (*p* = 0.31), emotional functioning (*p* = 0.14), or global QoL (*p* = 0.085).

Kenne et al. (2017) reported significant reductions in global distress (*p* = 0.013) and psychological symptoms (*p* = 0.019), together with improvements in mental health (*p* = 0.001), coping strategies (*p* = 0.028), and mindfulness.

Mondo et al. (2015) found significant increases in mindfulness (*p* = 0.024) and attentional focus, although self-esteem did not improve significantly (*p* = 0.203).

Franco et al. (2020) reported significant gains in resilience (Z = 2.97; *p* = 0.004) and self-esteem (Z = 5.06; *p* < 0.001).

Overall, mindfulness-based interventions appeared to improve psychological well-being and coping resources more consistently than global QoL indicators.

### Contextual cognitive-Behavioural and positive psychology interventions

Vila et al. (2016) observed significant reductions in emotional distress (Z = −4.149; *p* < 0.001), although no significant changes were found in anger (*p* = 0.345).

Cerezo et al. (2014) reported significant improvements in cognitive well-being, positive affect, negative affect, attention, and self-esteem (all *p* < 0.05).

Belber-Gómez et al. (2018) documented qualitative improvements in physical well-being, emotional validation, and adaptive coping.

Villoria et al. (2020) reported significant improvements in domestic activities (*p* = 0.005) and leisure participation (*p* = 0.05), although no significant improvements were observed in sexual activity or pleasurable activities.

Overall, these interventions appeared particularly useful for enhancing emotional adjustment, self-esteem, coping resources, and participation in daily activities.

### Yoga-based and self-administered interventions

Micheletti et al. (2022) reported short-term improvements in emotional functioning but no significant changes in global QoL or fatigue.

Chandwani et al. (2014) observed improvements in physical QoL (*p* = 0.05), psychological distress (*p* = 0.04), and fatigue reduction (*p* = 0.02), although no significant improvements were found in mental health (*p* = 0.20) or sleep quality (*p* = 0.90).

Hoogland et al. (2018) reported significant gains in emotional well-being (*p* = 0.01), functional well-being (*p* = 0.05), and physical well-being (*p* < 0.0001).

Overall, yoga-based and self-administered interventions appeared to contribute more consistently to physical functioning, fatigue reduction, and selected aspects of well-being than to broad improvements in global quality of life.

Across all intervention types, improvements were more consistently observed in emotional outcomes (anxiety, depression, distress) than in global quality-of-life measures (Table 6).

**Table 6:** Results.

Author and year	Design	Number of sessions	Sample	Techniques used (IV)	Measurement instruments	Results	Primary outcome	Multiple outcomes without predefined primary endpoint
Ardizzone et al. (2022)	Prospective RCT	IG: 3 individual 50-min sessions; Duration: 2 months	*N* = 60; IG: 30, CG: 30; Age: 35 to 70 years Italy	CBT + psychoeducational training; CG: self-guided informational booklet and individual counseling, no psychological treatment	[1] HADS; [2] Distress Thermometer (DT); [3] EORTC QLQ-C30 (Italian version 3.0)	Improvements in anxiety, depression, distress, overall QoL, physical, emotional, cognitive, and social functioning. Symptom reduction in fatigue, nausea, pain, dyspnea, insomnia. No significant differences between IG and CG after two months. Effect size: Not reported by authors.	No	Yes
Belber-Gómez et al. (2018)	Descriptive qualitative methodology	10 weekly 90-min group sessions	*N* = 6 women; Age: 35–64 Spain	Group ACT + Mindfulness	Survey to measure the level of satisfaction with the treatment.	Normalization of cancer coping process. Shift in disease perspective. Improved body image. Effect size: Not applicable (qualitative design).	No	Yes
Bisseling et al. (2017)	Mixed-method (quantitative and qualitative), uncontrolled cohort	8 weekly 2.5 h sessions + 6 h silent retreat + 45 min daily home practice	*N* = 64; Age: >18 years old Netherlands	Mindfulness-based stress reduction (MBSR) adapted for oncology, grief psychoeducation, optional partner sessions	[1] HADS [2] EORTC QLQ-C30 [3] Warwick-Edinburgh Mental Wellbeing Scale (WEMWBS). [4] Short Form Five Facet Mindfulness Questionnaire (FFMQ-SF). [5] Short Form Self-Compassion Scale (SCS-SF). [6] Ruminative Response Scale (RRS-BR).	Significant reduction in psychological distress. Improvements in mindfulness, self-compassion, wellbeing, physical/emotional functioning. No significant changes in rumination or overall QoL. Effect size: Not reported by authors (despite adequate sample size).	Unclear	Yes
Cerezo et al. (2014)	RCT	14 weekly 2 h sessions	*N* = 175; IG:87, Waitlist CG:88; Age: >18 Spain	Psychoeducation, mindfulness, positive communication, role-playing, humor	[1] Satisfaction with Life Scale [2] Affectivity Scale [3] TMMS-24. [4] LOT-R. [5] CD-RISC. [6] Rosenberg Self-Esteem Scale	The intervention group (GI) showed significant improvements in self-esteem (η^2^ = 0.05), optimism (η^2^ = 0.19), resilience (η^2^ = 0.09), well-being (η^2^ = 0.15), and happiness (phi = 0.47).	No	Yes
Chandwani et al. (2014)	RCT	3 weekly 60-min yoga sessions for 6 weeks (max. 18 sessions)	*N* = 163; YG:53, Stretching:56, CG:54; Age: >18 United States of America	Integrated yoga: asanas, pranayama, deep relaxation, meditation	[1] SF-36 [2] Brief Fatigue Inventory. [3] Pittsburgh Sleep Quality Index (PSQI). [4] Centers for Epidemiological Studies-Depression (CES-D). [5] Salivary cortisol	Yoga group showed better physical QoL and fatigue reduction. Positive effect on cortisol profile. Benefits maintained 6 months post-radiotherapy. Effect size: Not reported. The study only reported an *a priori* effect size for the sample size calculation (0.63 SD; based on a previous study reporting effect sizes of 0.44–0.47).	Yes (PCS of the SF-36)	Yes
Franco et al. (2020)	Quasi-experimental clinical trial, non-randomized CG	7 weekly 2-h sessions	*N* = 36; IG: 19, CG: 17; Age: 18–70 Spain	Instructor-led Mindfulness and Flow Meditation	[1] Acceptance and Action Questionnaire. (AAQ). [2] Social Avoidance and Distress Scale. [3] Profile of Mood States. [4] Connor-Davidson Resilience Scale. [5] Rosenberg Self-Esteem Questionnaire. [6] Daily log.	Significant reduction in anxiety and depression in IG. Improvement in physical, psychological, social QoL domains. No significant changes in anger, vigor, or fatigue. Effect size: (Cohen’s *d*): Depression = 1.22; Self-esteem = 1.17; Social avoidance = 1.03; Tension = 0.963; Experiential avoidance = 0.947; Resilience = 0.896; Social anxiety = 0.864; Fatigue = 0.441; Vigor = 0.334; Anger = 0.271.	No	Yes
González et al. (2015)	Pre-experimental, quantitative	10 individual 60-min sessions; Frequency: every 2–4 weeks	*N* = 15; Age: 31–67 Mexico	CBT	[1] HADS; [2] CAEPO; [3] WHOQOL-BREF	CBT improved QoL, reduced anxiety, and maladaptive coping. No reduction in depression, psychological health, or environment. Effect size: Large effects were observed for quality of life in the domains of physical health (Cohen’s d = −1.46), psychological health (d = −0.94), and interpersonal relationships (d = −1.15), as well as for depression (d = 0.98) and the overall HADS score (d = 1.50). Regarding coping strategies, the largest effect was found for reduced passive coping and passive regression (d = 0.34), whereas the remaining coping dimensions showed small effects (d = 0.19–0.24). Note: Negative effect sizes for the WHOQOL-BREF reflect the direction of score calculation and correspond to improvements in quality of life.	No	Yes
Hoogland et al. (2018)	RCT	No live sessions	*N* = 240; IG:121, CG:119; Age: >18 United States of America	Self-administered stress management (breathing, PMR, imagery, coping statements)	[1] HADS [2] Impact of Events Scale-Revised (IES-R). [3] FACT-G. [4] FACIT-SP. [5] Memorial Symptom Assesment Scale-Short form (MSAS-SF). [6] Mesure of Current Status (MOCS).	No significant group differences in emotional wellbeing. IG increased use of stress management techniques significantly. Effect size: No group differences were observed for primary or secondary outcomes (all Cohen’s ds < 0.05). A moderate effect was found for increased use of stress management techniques (d = 0.42).	Yes. Physical Component Summary (PCS) of the SF-36.	Yes
Kenne et al. (2017)	RCT, longitudinal (3-month follow-up)	8 weekly 2 h sessions + daily mindfulness practice	*N* = 166; IG:62, active CG:52, CG:52; Age: 34–80 Sweden	Standard MBSR	[1] HADS [2] Memorial Symptom Assessment Scale (MSAS). [3] SF-36. [4] Sense of Coherence Scale (SOC). [5] Five Facets Mindfulness Questionnaire (FFMQ). [6] Posttraumatic Growth Inventory (PTGI). [7] Inmune biomarkers: NK cells, IL-6, IL-8	Sustained psychological and biological benefits in breast cancer patients. Significant improvements in depression, symptoms, vitality, mental health, PTG in IG. NK cell activity and immune response improved. No significant change in anxiety. Effect size: Not reported by authors (biological and psychological outcomes reported as significant but without standardized effect indices).	Yes. Anxiety and depression (HADS)	Yes
Martínez-Cuervo et al. (2020)	Pre-experimental study	6 sessions of 120 min each; 6-month follow-up	*N* = 17; Mean age: 52 Mexico	Group CBT with psychoeducation, relaxation, cognitive restructuring	[1] HTD8808 Digital Thermometer. Assesses nasal peripheral temperature. [2] Beck Depression Inventory (BDI). Measures depressive symptoms. [3] Hamilton Anxiety Scale (HAM-A). Measures the intensity of anxiety symptoms.	Significant decrease in depression and anxiety (*p* < 0.05). Decrease in nasal peripheral temperature. Effect size: reported large effect sizes following a group-based CBT programme, with significant reductions in anxiety (partial η^2^ = 0.254) and depression (partial η^2^ = 0.274).	Yes. Depression and anxiety (BDI, HAM-A)	No
Micheletti et al. (2022)	RCT	10 sessions (2x/week) over 5 weeks; 75 min/session	*N* = 24; IG:12, CG:12; Age: >18 Italy	Classical yoga adapted for radiotherapy patients	[1] SF-36. [2] EORT QLQ-C30 [3] Brief Fatigue Intervention (BFI) [4] Pittsburgh Sleep Quality Index (PSQI) [5] Blood cortisol & inflammatory biomarkers (IL-6, IL-10, IL-1RA, TNF-α, LMR)	Significant improvement in emotional and physical QoL. Decrease in cortisol levels and positive trend in inflammation (not all biomarkers). Effect size: Not reported by authors.	No	Yes
Mondo et al. (2015)	Pilot quasi-experimental study (no CG)	8 sessions	*N* = 7; Age: 40–68 Spain	MBCT (Segal protocol)	[1] HADS; [2] Rosenberg Self-Esteem; [3] EMPE	Significant reduction in depression; trend toward reduced anxiety. Increased mindfulness and awareness. No change in self-esteem. Effect size: Not reported by authors.	Unclear	Yes
Moraga et al. (2020)	Longitudinal quasi-experimental pre-post design with post-test only CG	G1: 10 individual sessions (7 months); G2: psychoeducation and counseling (7 months)	*N*: 180 G1:50 G2:98 CG:32 Mean age: 51.9 Spain	Three groups: G1: clinical symptoms, CBT + psychoeducation; G2: psychoeducation + counseling; CG: post-test only after 7 months	[1] HADS [2] Test Mini Mental Adjustment to Cancer (MINI-MAC) of Watson.	CBT more effective than counseling + psychoeducation in reducing anxiety and depression. Counseling helped maintain fighting spirit and non-clinical anxiety/depression levels. Effect size: Large effects were observed for depression (partial η^2^ = 0.274), anxiety (partial η^2^ = 0.254), hopelessness (partial η^2^ = 0.202), and anxious preoccupation (partial η^2^ = 0.150). A moderate effect was found for fighting spirit (partial η^2^ = 0.111). In contrast, cognitive avoidance (partial η^2^ = 0.001) and fatalism (partial η^2^ < 0.001) showed negligible effect sizes and no significant changes over time.	No	Yes
Vila et al. (2016)	Empirical quantitative descriptive study	Single session; 3 timepoints (last week, before and after visit)	*N* = 30; Age: 27–83 Spain	Problem-solving, psychoeducation, cognitive restructuring, mindfulness, acceptance, visualization, positive aspects	[1] Distress Thermometer (DT)	Reduction in sadness, nervousness, fear, and emotional disturbance post-visit. Statistical significance was reported for emotional distress reduction. Effect size: Not reported by authors.	No	Yes
Villoria et al. (2020)	Descriptive and statistical study of clinical change	6 individual 45-min sessions; 3-month follow-up post-chemotherapy.	*N* = 18; Mean age: 49.22 Spain	Contingency contracts, skill training, metaphors	[1] HADS; [2] Semi-structured interview	Functional improvements across life domains. Significant recognition and reduction in avoidance behaviour. Mood improved but not statistically significant (assessed with HADS). Effect size: Not reported by authors.	No	Yes

IG = Intervention Group; CG = Control Group; QoL = Quality of Life; ACT = Acceptance and Commitment Therapy; MBSR = Mindfulness-Based Stress Reduction; MBCT = Mindfulness-Based Cognitive Therapy; CBT = Cognitive Behavioural Therapy; PMR = Progressive Muscle Relaxation.

CBT interventions produced the most robust and consistent reductions in anxiety and depression. Mindfulness-based interventions showed moderate benefits for emotional distress and psychological well-being. Other psychosocial intervention approaches yielded more heterogeneous outcomes. Positive psychology interventions showed notable improvements in well-being, self-esteem, positive affect, and resilience, alongside reductions in sadness. These benefits were observed in both brief, single-session formats and more extended interventions, underscoring the potential of such approaches to strengthen personal resources during treatment.

### Anxiety and depression

Anxiety and depression were among the most frequently evaluated variables, appearing in 11 and 12 studies, respectively. Overall, most trials reported significant reductions in both outcomes, particularly following CBT-based and mindfulness-based interventions. Martínez-Cuervo et al. (2020) and Moraga et al. (2020), who implemented group and individual CBT respectively, found significant reductions in anxiety and depression. The former reported large effect sizes, with significant decreases in anxiety (*F* = 49.66; *p* < 0.001; partial η^2^ = 0.254) and depression (*F* = 55.24; *p* < 0.001; partial η^2^ = 0.274). The latter study showed significant pre-post reductions in anxiety (t = 4.61; *p* = 0.001) and depression (t = 2.99; *p* = 0.013), suggesting meaningful improvements despite not reporting direct effect-size indices. Ardizzone et al. (2022), who compared brief CBT with an informational guide, similarly observed significant reductions in anxiety (t = 3.72; *p* = 0.000) and depression (t = 3.13; *p* = 0.003). González et al. (2015) also reported a significant decrease in anxiety (t = −2.36; *p* = 0.027), although depression did not show statistically significant change (t = 1.84; *p* = 0.08); however, significant improvements appeared in the global HADS score (T = 2.81; *p* = 0.014).

Although Vila et al. (2016) did not directly assess anxiety or depression, they evaluated related emotional states and found significant reductions in sadness intensity (Z = −3.239; *p* = 0.001), nervousness (Z = −3.030; *p* = 0.002), fear (Z = −2.416; *p* = 0.016), and emotional disturbance (Z = −3.030; *p* = 0.002) after a single psycho-oncological session based on counselling and positive psychology techniques. Results from third-wave interventions were more heterogeneous. In studies using Mindfulness-Based Stress Reduction (MBSR), Bisseling et al. (2017) reported no significant improvements in HADS scores (*p* = 0.512), whereas Kenne et al. (2017) observed significant reductions (*p* = 0.003). Mondo et al. (2015) documented significant reductions in depression (Z = −2.120; *p* = 0.034) and a marginal reduction in anxiety (Z = −1.807; *p* = 0.071). Franco et al. (2020), using flow meditation, found significant improvements across several outcomes, including anxiety (Z = 2.68; *p* = 0.009), social avoidance (Z = 3.57; *p* = 0.001), and depression (Z = 4.56; *p* < 0.001).

In contrast, self-administered interventions produced weaker results. Hoogland et al. (2018) demonstrated significant improvements in stress management (*p* < 0.05) but found no significant group differences in anxiety or cancer-related distress. Among yoga-based studies, only Chandwani et al. (2014) assessed depression, with no clear improvements in anxiety or depression reported. Finally, Villoria et al. (2020), using Acceptance and Commitment Therapy (ACT), did not obtain significant changes in either anxiety or depression.

### Quality of life and well-being

An important observation was that emotional outcomes were assessed more frequently and consistently than multidimensional quality-of-life measures. While anxiety and depression were evaluated in most studies, only a subset of interventions examined broader QoL domains using standardized instruments. Quality of life (QoL) and well-being are often treated as related constructs, yet not all studies assessed them equally; therefore, in Table 4, these variables have been presented separately. The results concerning these outcomes are summarized below. Among trials employing CBT, González et al. (2015) reported significant improvements in the QoL subscales of physical health (*p* = 0.016) and interpersonal relationships (*p* = 0.048), although no significant changes were observed in general psychological health (*p* = 0.099) or environmental well-being (*p* = 0.567). Ardizzone et al. (2022), using the EORTC QLQ-C30, found significant pre-post improvements in global QoL (*p* < 0.001) and in physical, emotional, and social functioning (all *p* < 0.001), as well as significant reductions in fatigue, nausea, pain, dyspnea, and insomnia (all *p* < 0.001); however, no significant between-group differences were reported.

By contrast, studies involving mindfulness and other third-wave therapies produced mixed findings. Vila et al. (2016) observed immediate reductions in emotional distress (Z = −4.149; *p* < 0.001) following a single positive-psychology-based psychotherapy session, although no significant change was noted in anger (*p* = 0.345). Cerezo et al. (2014), using a longer group-based positive psychology intervention, reported significant improvements in cognitive well-being, positive and negative affect, attention, and self-esteem (all *p* < 0.05). Belber-Gómez et al. (2018) documented qualitative improvements in physical well-being, emotional validation, and adaptive coping strategies using ACT. Villoria et al. (2020), also employing ACT, reported significant improvements in domestic activities (*p* = 0.005) and increased leisure participation (*p* = 0.05), but no improvements in sexual activity or pleasurable activities.

Mindfulness-Based Stress Reduction (MBSR) interventions yielded similarly variable outcomes. Bisseling et al. (2017) did not observe significant improvements in physical well-being (*p* = 0.31), emotional functioning (*p* = 0.14), or global QoL (*p* = 0.085). In contrast, Kenne et al. (2017) reported significant reductions in global distress (*p* = 0.013) and psychological symptoms (*p* = 0.019), along with improved mental health (*p* = 0.001), coping strategies (*p* = 0.028), and mindfulness. Mondo et al. (2015) found increased mindfulness (*p* = 0.024) and greater attentional focus, although self-esteem did not significantly improve (*p* = 0.203). Hoogland et al. (2018), evaluating a self-administered stress-management program, reported significant gains in emotional (*p* = 0.01), functional (*p* = 0.05), and physical well-being (*p* < 0.0001). Franco et al. (2020), using Flow Meditation, documented significant increases in resilience (Z = 2.97; *p* = 0.004) and self-esteem (Z = 5.06; *p* < 0.001), both closely related to overall well-being. Notably, only a limited number of mindfulness-based studies met the eligibility criteria for inclusion in this review. This finding contrasts with the broader prominence of mindfulness within psycho-oncology and appears to reflect the relatively small number of studies conducted exclusively during active treatment.

Findings from yoga-based interventions were also inconsistent. Micheletti et al. (2022) observed only short-term improvements in emotional functioning, with no significant changes in global QoL or fatigue. In contrast, Chandwani et al. (2014) reported improvements in physical QoL (*p* = 0.05), psychological distress (*p* = 0.04), and a significant reduction in fatigue (*p* = 0.02), though no significant improvements were found in mental health (*p* = 0.20) or sleep quality (*p* = 0.90).

## Discussion

The present review suggests that emotional distress may have a particularly strong impact during active treatment because patients simultaneously face uncertainty regarding treatment response and substantial physical burden. This systematic review examined the influence of psychosocial factors on the quality of life (QoL) of women undergoing active treatment for breast cancer, as well as the effectiveness of various psychological interventions aimed at improving emotional well-being and broader QoL outcomes. Overall, the findings indicate that QoL in oncology patients is closely linked to emotional variables—particularly anxiety and depression—while also being shaped by social and relational factors such as coping strategies, family support, and perceptions of the surrounding environment. Notably, evidence supporting improvements in emotional symptoms was considerably stronger than that for global, multidimensional QoL indices. Although several interventions produced statistically significant reductions in anxiety and depression, improvements in overall QoL were less consistent and frequently restricted to specific subdomains—such as physical or social functioning—rather than reflected in comprehensive composite scores. This pattern is consistent with previous psychosocial oncology research indicating that psychological interventions during active cancer treatment tend to produce stronger effects on emotional distress than on broader multidimensional quality-of-life outcomes. Previous reviews have similarly reported that reductions in anxiety, depression, and cancer-related distress often precede measurable improvements in global QoL indices, particularly during intensive treatment phases. One possible explanation is that global QoL during active treatment is strongly influenced by ongoing physical symptoms, treatment schedules, fatigue, pain, financial concerns, and disruptions to work and family life. Consequently, psychological interventions may successfully improve emotional adjustment without producing immediate changes in broader multidimensional QoL indicators.

Cognitive-behavioural therapy (CBT)-based interventions, as reported by Ardizzone et al. (2022), Martínez-Cuervo et al. (2020), and Moraga et al. (2020), demonstrated significant reductions in anxiety and depression, reinforcing their clinical utility during active oncological treatment. These findings are in line with the broader psychosocial oncology literature, which consistently identifies CBT as one of the most empirically supported interventions for managing emotional distress among cancer patients. However, results were not uniformly consistent across studies. For example, González et al. (2015) documented reductions in anxiety and improvements in physical and social QoL domains, but found no statistically significant changes in depression or overall psychological health. Even so, significant improvements on the general Hospital Anxiety and Depression Scale (HADS) suggested some degree of depressive symptom alleviation, although not independently significant. These discrepancies may reflect limitations such as the small sample size (*N* = 15) or the absence of a control group.

Mindfulness-based and other psychosocial interventions yielded more heterogeneous outcomes. Positive psychology–based interventions showed notable improvements in well-being, self-esteem, positive affect, and resilience, alongside reductions in sadness. These benefits were observed in both brief, single-session formats (Vila et al., 2016) and more extended interventions (Cerezo et al., 2014), underscoring the potential of such approaches to strengthen personal resources during treatment. Mindfulness-Based Stress Reduction (MBSR) programs (Bisseling et al., 2017; Kenne et al., 2017; Mondo et al., 2015) consistently reported emotional benefits, including mood enhancement, increased mindfulness, and reductions in distress. The relatively small number of mindfulness-based studies identified in this review is noteworthy given the broader prominence of mindfulness within psycho-oncology. This finding likely reflects the current distribution of the literature rather than a lack of interest in mindfulness-based approaches. A substantial proportion of mindfulness research in breast cancer has been conducted with survivors or mixed oncology populations, whereas comparatively fewer studies have focused exclusively on women undergoing active treatment. Consequently, additional high-quality studies are needed to clarify the role of mindfulness-based interventions during active breast cancer treatment. However, findings concerning global QoL were variable: some interventions yielded significant improvements (Bisseling et al., 2017), whereas others produced gains limited to mental health and functional capacity (Kenne et al., 2017). Similarly, Flow Meditation—a mindfulness-based intervention applied by Franco et al. (2020)—produced decreases in anxiety and depression and increases in resilience and self-esteem, all factors closely tied to QoL. These results collectively suggest that mindfulness-based therapies are particularly effective for alleviating emotional distress, although their capacity to improve overall QoL remains less clear. Future research should therefore delineate which QoL dimensions are most responsive to mindfulness interventions.

Findings regarding behavioural activation were more modest. Villoria et al. (2020) did not observe significant reductions in anxiety or depression; however, improvements in functional capacity and decreases in avoidant behaviours were documented. Belber-Gómez et al. (2018) reported similar qualitative improvements—such as enhanced body image and coping skills—but did not provide quantitative indicators of clinical efficacy. These results suggest that behavioural activation may facilitate behavioural adjustment rather than producing immediate changes in emotional symptomatology.

Studies examining yoga-based interventions also produced mixed findings. Chandwani et al. (2014) reported significant improvements in physical QoL and reductions in fatigue, whereas Micheletti et al. (2022) observed only short-term benefits without notable improvements in global QoL or fatigue levels. Variability in intervention duration, yoga modality, and timing within the treatment trajectory may account for these inconsistencies.

A self-administered stress-management intervention (Hoogland et al., 2018) resulted in improvements in emotional, functional, and physical well-being; however, no significant between-group differences emerged for anxiety or overall QoL. These findings highlight the importance of professional supervision in optimizing psychological intervention outcomes, particularly for patients undergoing highly demanding medical treatments.

Taken together, the evidence suggests that psychological interventions play a meaningful role in supporting emotional adjustment during active breast cancer treatment. Nevertheless, the methodological heterogeneity across studies, high risk of bias in several domains, and reliance on short-term assessments introduce important limitations. As such, conclusions regarding sustained improvements in global QoL must be interpreted with caution. More rigorous, longitudinal randomized controlled trials are required to establish the durability, generalizability, and clinical significance of these effects over time.

Another methodological issue concerns multiple statistical testing. Several included studies evaluated numerous psychological and quality-of-life outcomes while reporting multiple statistical comparisons without consistently identifying predefined primary endpoints or applying corrections for multiple testing. Consequently, some statistically significant findings may reflect an increased probability of Type I error, particularly in studies with relatively small samples. These issues further support cautious interpretation of the reported intervention effects.

Importantly, only seven of the fifteen included studies explicitly assessed QoL using standardized instruments such as the EORTC QLQ-C30 or the SF-36, and findings regarding significant improvements were mixed. Such inconsistencies may be attributable to differences in measurement tools, variability in intervention modalities, or disparities in intervention duration and timing relative to treatment.

Overall, the findings of this review align with a growing body of psychosocial oncology research highlighting the importance of integrating psychological care into oncology treatment pathways. Emotional distress—particularly anxiety and depressive symptoms—has repeatedly been identified as one of the most significant determinants of patient-reported quality of life during cancer treatment. Consequently, interventions targeting emotional regulation, coping strategies, and psychological flexibility appear especially relevant during active treatment phases.

An important implication of these findings concerns the implementation of psychosocial interventions during active treatment. Women undergoing chemotherapy, radiotherapy, surgery, or hormone therapy frequently experience fatigue, symptom burden, demanding treatment schedules, and disruptions to everyday responsibilities. These factors may reduce attendance, adherence, and engagement in psychosocial programs, regardless of their potential effectiveness. Consequently, intervention feasibility should be considered alongside efficacy when evaluating psychosocial care during active treatment.

The findings of this review suggest that brief interventions, professionally supported programs, and flexible delivery formats may be particularly suitable for this population. Telehealth interventions, hybrid delivery models, and stepped-care approaches may help reduce participation barriers while preserving therapeutic benefits. Future studies should therefore incorporate feasibility, acceptability, treatment burden, and adherence outcomes in addition to traditional efficacy measures.

### Limitations and strengths

A particular strength of this review is its exclusive focus on women undergoing active breast cancer treatment, a population that has frequently been combined with survivorship samples in previous reviews despite the distinctive clinical and psychosocial demands associated with ongoing treatment. This systematic review presents several limitations. First, heterogeneity across study designs, QoL and well-being instruments, and the psychosocial variables assessed hindered standardized comparisons and complicated the extraction of robust and generalizable conclusions. Second, many studies were characterized by small sample sizes and the absence of control groups, which further limits the external validity of the findings. Additionally, some interventions—such as those described in Hoogland et al. (2018)—were self-administered without therapist supervision, a factor that may have compromised their effectiveness. A further limitation lies in the predominance of short-term assessments, which restricts understanding of the long-term sustainability of intervention effects. Collectively, these methodological constraints limit the capacity to formulate definitive conclusions and underscore the need for future research employing unified criteria, larger samples, and more rigorous designs.

This review also presents certain methodological limitations. The search strategy was restricted to specific databases and publications in English or Spanish, which may have led to the omission of relevant studies. In addition, heterogeneity in study designs and outcome measures limited the possibility of performing quantitative synthesis. An additional limitation concerns the inconsistent reporting of primary and secondary outcomes across the included studies. Many studies assessed multiple psychological and quality-of-life variables without clearly specifying primary endpoints, which complicated comparisons between interventions and restricted the ability to identify the most clinically relevant treatment effects.

An additional methodological limitation of the available evidence is that most studies did not prespecify a primary outcome and frequently assessed multiple psychological endpoints without adjustment for multiple testing. This increases the likelihood of false-positive findings and limits confidence in isolated statistically significant results, particularly in studies with small sample sizes.

Furthermore, although several studies reported standardized effect sizes, this practice was inconsistent across the included studies, and confidence intervals were seldom reported. This limited the ability to consistently evaluate the magnitude, precision, and clinical relevance of intervention effects beyond statistical significance.

Despite these limitations, the review demonstrates notable strengths. The findings highlight the value of integrating psychological interventions into oncology services as part of comprehensive patient care. In particular, CBT and mindfulness-based interventions—especially when delivered briefly and under professional supervision—consistently yielded improvements in emotional well-being and, in some cases, broader QoL domains among women undergoing active breast cancer treatment. These results reinforce the relevance of adopting a biopsychosocial framework in oncology that addresses medical, psychological, and relational aspects of patient well-being. Future studies should prioritize longitudinal methodologies and robust experimental designs to clarify causal mechanisms and optimize the implementation of these interventions in clinical practice. This observation is consistent with previous studies in psycho-oncology suggesting that mindfulness-based approaches primarily influence emotional regulation, stress reduction, and acceptance processes, while their effects on broader quality-of-life constructs may emerge more gradually over time.

Another methodological limitation is that PubMed was not included among the searched databases. Although Web of Science, Scopus, ProQuest and Dialnet provide extensive coverage of psychological, psychosocial and multidisciplinary literature, PubMed is a core biomedical database that indexes a large proportion of psycho-oncology and oncology research. Consequently, potentially relevant studies may have been missed, and this should be considered when interpreting the comprehensiveness of the present review.

### Future research directions

The findings of this review identify several key priorities for future research. Future research should prioritize adequately powered randomized controlled trials employing standardized and comparable QoL instruments to improve cross-study comparability and reduce methodological heterogeneity. Longer follow-up periods are also required to determine whether improvements in emotional well-being translate into sustained benefits across broader QoL domains during and after active treatment. Future studies should clearly distinguish between emotional symptom outcomes (e.g., anxiety and depression) and multidimensional QoL constructs at both the conceptual and analytical levels. This distinction may help clarify why interventions often demonstrate stronger effects on emotional distress than on global QoL indicators during active treatment.

Research should further investigate potential mechanisms of change, including psychological flexibility, coping strategies, resilience, mindfulness skills, and social support, in order to better understand how different intervention approaches exert their effects.

Importantly, future studies should also address practical aspects of psychosocial intervention delivery during active treatment, including feasibility, patient adherence, treatment burden, accessibility, and integration into routine oncology care. Research evaluating brief interventions, telehealth programs, hybrid delivery models, and stepped-care approaches may be particularly valuable given the physical and logistical challenges experienced by many patients during chemotherapy and radiotherapy.

Finally, comparative trials conducted under similar methodological conditions and directly contrasting different psychosocial intervention approaches—including CBT, mindfulness-based interventions, ACT, behavioural activation, positive psychology interventions, and supportive care programs—would be particularly valuable in determining whether specific approaches demonstrate greater effectiveness for particular psychosocial domains or treatment stages.

First, adequately powered randomized controlled trials using standardized and comparable QoL instruments are needed to reduce measurement heterogeneity and enhance cross-study comparability. Second, longer follow-up periods are essential to determine the durability of psychological intervention effects beyond short-term symptom reduction, thereby clarifying whether observed benefits persist throughout the treatment trajectory and into survivorship. Third, future studies should clearly distinguish between emotional symptom outcomes—such as anxiety and depression—and multidimensional global QoL constructs during both study design and statistical analysis, as this conceptual differentiation would allow for more precise interpretation of intervention efficacy. Fourth, research should examine potential mediators and mechanisms of change, including psychological flexibility, coping strategies, resilience, and social support, to better elucidate how distinct therapeutic approaches exert their effects during active oncological treatment. Finally, comparative trials conducted under similar methodological conditions and directly contrasting second-generation interventions with third-wave therapies would be particularly valuable in determining whether specific approaches demonstrate greater effectiveness for particular psychosocial domains or treatment stages. Addressing these areas would substantially advance the theoretical refinement, methodological rigor, and clinical applicability of psychosocial interventions in breast cancer care. Finally, comparative trials conducted under similar methodological conditions and directly contrasting different psychosocial intervention approaches—including CBT, mindfulness-based interventions, ACT, behavioural activation, and positive psychology interventions—would be particularly valuable in determining whether specific approaches demonstrate greater effectiveness for particular psychosocial domains or treatment stages.

## Conclusion

The available evidence suggests that cognitive-behavioural therapy (CBT), together with several psychosocial intervention approaches—including mindfulness-based interventions, Acceptance and Commitment Therapy, positive psychology interventions, and related supportive programmes—may contribute to short-term reductions in anxiety and depressive symptoms among women undergoing active breast cancer treatment. Evidence for improvements in specific quality-of-life dimensions—particularly emotional and social functioning—is promising yet remains heterogeneous across studies. At present, the available findings do not permit firm conclusions regarding sustained or global QoL enhancement, nor do they clarify whether specific psychosocial intervention approaches are more effective than others for particular emotional or quality-of-life outcomes. Importantly, the active treatment phase presents unique physical, emotional, and practical challenges that may influence both the effectiveness and feasibility of psychosocial interventions, highlighting the need for treatment-phase-specific evidence and implementation strategies. However, these findings should be interpreted cautiously given the limited number of studies, relatively small sample sizes, methodological heterogeneity, inconsistent reporting of effect sizes, and the overall risk of bias across the included studies. Therefore, adequately powered randomized controlled trials employing standardized outcome measures, predefined primary endpoints, comprehensive effect-size reporting, and longer follow-up periods are needed before firm conclusions can be drawn regarding the comparative effectiveness and clinical relevance of different psychosocial interventions during active breast cancer treatment.
